# Effect of dexmedetomidine, lignocaine and combination infusion for postoperative outcomes in abdominal surgery

**DOI:** 10.6026/973206300214621

**Published:** 2025-12-15

**Authors:** Monika Gandhi, Shreya Chourasia, Aseem Sharma, Shailendra Singh, Shalini Jain

**Affiliations:** 1 Department of Anaesthesiology, M.G.M. Medical College & M.Y. Hospital, Indore- 452001, Madhya Pradesh, India; 2 Department of Emergency Medicine, M.G.M. Medical College & M.Y. Hospital, Indore- 452001, Madhya Pradesh, India

**Keywords:** Neonatal jaundice, parental judgement, Kramer's scale, serum bilirubin, hyperbilirubinaemia

## Abstract

Acute postoperative pain causes stress response and delays patient recovery after abdominal surgery. Therefore, it is of interest to evaluate the effect of intravenous dexmedetomidine, lignocaine and their combination on postoperative pain and recovery under general anesthesia. Ninety patients undergoing abdominal surgery were observed and assessed using NRS and QoR-15 at 24 and 48 hours. Combination group showed lowest pain score, least analgesic use and best recovery indicating superior analgesic benefit with combined use.

## Background:

Postoperative pain is still one of the main reasons for poor patient satisfaction and delayed discharge after major abdominal surgeries. It leads to stress response, delayed mobilization and sometimes chronic pain. Effective perioperative analgesia is the key for better recovery and shorter hospital stay [1, 2- 3]. Dexmedetomidine, an alpha-2 agonist, and lignocaine, a sodium channel blocker, both have analgesic and anti-inflammatory properties with minimal respiratory depression [4, 5- 6]. Therefore, it is of interest to evaluate the effect of dexmedetomidine, lignocaine and combination infusion for postoperative outcomes in abdominal surgery.

## Materials and Methods:

This prospective observational study was conducted in a tertiary care hospital in Central India from September 2023 to October 2024 after approval from the Institutional Ethics Committee. Ninety adult patients aged 18-65 years of either sex, belonging to ASA I and II, undergoing elective abdominal surgery under general anaesthesia were included after written informed consent. According to the intraoperative use of drugs, patients were observed in three groups - those receiving dexmedetomidine, lignocaine, or a combination of both as part of standard anaesthetic practice. In Group D, dexmedetomidine 1 mcg/kg loading over 15 min was given followed by 0.5 mcg/kg/h infusion. In Group L, lignocaine 1.5 mg/kg loading over 15 min followed by 1 mg/kg/h infusion was used. In Group DL, both drugs were given together at the same doses till the end of surgery. Anaesthesia was induced with fentanyl 2 mcg/kg, propofol 2 mg/kg, and atracurium 0.5 mg/kg. Pain intensity was assessed by Numeric Rating Scale (NRS) [7, 8] at 0, 1, 2, 4, 8, 12, 18 and 24 hours after surgery. When NRS > 4, intravenous diclofenac 75 mg was administered as rescue analgesic. Quality of recovery was assessed using QoR-15 score [9, 10, 11-12] at 24 and 48 hours. Data were analyzed using SPSS version 22. Categorical data were compared with Chi-square test and mean values by one-way ANOVA followed by Tukey post-hoc test. A P value < 0.05 was considered statistically significant.

## Results:

Ninety patients were analysed in three groups (D, L, and D+L). Postoperative pain (NRS) differed significantly between groups at most time points (ANOVA p = 0.001) with the combination group showing lower scores at 0 min (1.83±0.70 vs 2.50±0.82 in D and 3.20±0.89 in L), 1 h (2.47±0.94 vs 3.00±0.74 and 5.17±0.83), and 2 h (2.83±0.59 vs 5.33±0.71 and 3.00±0.69), while no difference was seen at 4 h (p = 0.939), later differences again appeared at 8 h (p = 0.001) and 12 h (p = 0.001) with significant post-hoc separation mainly between D vs L and D vs D+L whereas L vs D+L was not significant at 8 h and 18 h (Table 1 - see PDF). Rescue analgesic consumption at 18 h and 24 h also varied significantly with higher NRS in L and lower in D+L at 24 h (1.97±0.72 in D+L vs 4.27±0.83 in D and 3.23±1.01 in L; p = 0.001) (Table 2 - see PDF). Total analgesic requirement in the first 24 h was lowest in the combination group (137.50±28.43 mg), intermediate in D (187.50±38.14 mg) and highest in L (247.50±34.96 mg) with significant post-hoc differences across comparisons (p = 0.001) (Table 3 - see PDF). Quality of recovery (QoR-15) was significantly better with the combination at both 24 h (123.83±3.23 vs 103.10±5.49 in D and 97.50±2.85 in L; p = 0.001) and 48 h (137.90±6.38 vs 129.57±4.15 and 122.17±4.49; p = 0.001), with all pairwise Tukey comparisons significant (Table 4 - see PDF).

## Discussion:

Among 90 patients included, demographic and intraoperative parameters were comparable between all groups. Mean postoperative pain scores assessed by NRS showed clear difference at most time intervals. Combination group (DL) had lowest mean NRS at 0, 1, 2, 12, 18 and 24 hours compared to dexmedetomidine (D) or lignocaine (L) alone (P < 0.05). At 4 hours no significant difference was noted suggesting transient convergence of analgesic effect. Total rescue analgesic requirement in first 24 hours was least in Group DL (137.50 ± 28.43 mg), followed by Group D (187.50 ± 38.14 mg) and highest in Group L (247.50 ± 34.96 mg) with P = 0.001. Quality of recovery score (QoR-15) showed consistent improvement with combination group at both 24 h (123.83 ± 3.23) and 48 h (137.90 ± 6.38) significantly higher than D or L groups (P < 0.001). These findings indicate better analgesia, lesser analgesic use, and faster recovery in patients receiving combined dexmedetomidine-lignocaine infusion. Our study shows a clear and consistent benefit with combined dexmedetomidine-lignocaine infusion for abdominal surgery patients. Pain scores were lowest in the combination group at early hours and again from 12-24 hours, with a small convergence around 4 hours. Rescue analgesic need was least with combination, and quality of recovery was highest at 24 and 48 hours. These findings point toward better analgesia, less opioid use, and faster recovery when both drugs are used together. Evidence from prior work supports this signal. Xu *et al.* reported lower pain scores and less opioid need when lidocaine and dexmedetomidine were given together after abdominal hysterectomy [13]. Guo *et al.* also showed improved pain control with the combination after thyroidectomy, adding external validity across a different surgical model [14]. Our results align with both studies, suggesting that the benefit is not limited to one operation type. Some reports did not find clear separation between the two single drugs. Cho *et al.* observed no significant difference in pain scores between lignocaine and dexmedetomidine after laparoscopic cholecystectomy [15]. Rekatsina *et al.* also found no difference between single-drug groups in gynecologic surgery [16]. Dosing schemes, surgery type, and background multimodal analgesia likely explain part of this heterogeneity, since higher or faster lignocaine infusion and shorter surgeries can flatten intergroup gaps. Mechanism wise, the pair seems complementary. Dexmedetomidine reduces central sympathetic drive and nociceptive transmission, which lowers perioperative analgesic need [17]. Lignocaine reduces peripheral input and central sensitization with anti-inflammatory effect, which helps late pain and bowel recovery [18]. Hitting both central and peripheral pathways together may explain the sustained benefit we saw after 12 hours, when single agents start to drift. The recovery data follow the same direction. QoR-15 was highest with the combination at both time points, matching earlier reports that linked better analgesia with better global recovery after surgery [19]. Sivaji *et al.* showed superior recovery scores with the combined infusion after robotic hysterectomy, which is consistent with our observation [20]. Between single agents, dexmedetomidine alone performed better than lignocaine for QoR, in line with findings by Kumari *et al.* where dexmedetomidine improved early recovery over lignocaine [21]. Analgesic consumption mirrored pain scores. Combination had the lowest 24-hour requirement, which is clinically meaningful for opioid-sparing pathways. Singh *et al.* also found lower tramadol use with dexmedetomidine versus lignocaine, supporting our single-agent ranking where dexmedetomidine outperformed lignocaine [22]. Together these data favor a step-up approach where the combination is preferred for high-pain abdominal procedures, and dexmedetomidine alone can be a reasonable second choice when combination is not feasible. We must interpret these results with care because the design is observational. Allocation followed routine practice, so residual confounding and selection bias cannot be fully excluded even when baseline factors look balanced. Unmeasured variables like surgical handling, intraoperative nociception, fluid and antiemetic strategy can influence pain and recovery. Our sample size is modest, and we did not run adjusted models for all potential confounders, so effect estimates may be conservative or inflated. Still the direction is consistent, the effect spans multiple endpoints and the pattern matches prior literature across different operations. Next step should be larger pragmatic trials or well-designed propensity-matched cohorts with standardized dosing windows, nociception-guided titration, and patient-centred recovery outcomes, to confirm durability, safety, and generalizability.

## Conclusion:

The combination of intravenous dexmedetomidine and lignocaine provided superior postoperative analgesia and recovery compared to either drug alone. Patients receiving both drugs had lower pain scores, reduced analgesic requirement, and higher QoR-15 scores. Dexmedetomidine alone was more effective than lignocaine but the combination offered the most consistent benefit. Hence the combined infusion may serve as a better perioperative strategy for abdominal surgeries.
